# Optically levitated micro gyroscopes with an MHz rotational vaterite rotor

**DOI:** 10.1038/s41378-024-00726-0

**Published:** 2024-06-18

**Authors:** Kai Zeng, Xiangming Xu, Yulie Wu, Xuezhong Wu, Dingbang Xiao

**Affiliations:** 1 College of Intelligence Science and Technology, National University of Defense Technology, Changsha, 410073 China; 2 College of Information and Communication, National University of Defense Technology, Wuhan, 430000 China; 3 Key Laboratory of Satellite Navigation Technology, National University of Defense Technology, Changsha, 410073 China

**Keywords:** Physics, Optical sensors

## Abstract

The field of levitated optomechanics has experienced significant advancements in manipulating the translational and rotational dynamics of optically levitated particles and exploring their sensing applications. The concept of using optically levitated particles as gyroscopes to measure angular motion has long been explored but has not yet been proven either theoretically or experimentally. In this study, we present the first rotor gyroscope based on optically levitated high-speed rotating particles. The gyroscope is composed of a micrometer-size ellipsoidal vaterite particle that is driven to rotate at MHz frequencies in a vacuum environment. When an external angular velocity is input, the optical axis deviates from its initial position, resulting in changes in the frequency and amplitude of the rotational signal. By analyzing these changes, the angular velocity of the input can be accurately detected, making it the smallest rotor gyroscope in the world. The angular rate bias instability of the gyroscope is measured to be 0.08°/s and can be further improved to as low as 10^−9^°/h theoretically by cooling the motion and increasing the angular moment of the levitated particle. Our work opens a new application paradigm for levitated optomechanical systems and may pave the way for the development of quantum rotor gyroscopes.

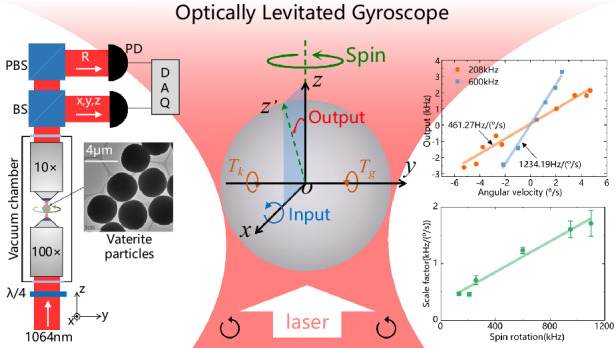

## Introduction

The optical levitation system employs a focused laser to trap particles, generating a gradient force that directs the particles toward the focal point. This enables a stable particle suspension without the need for additional control^1–3^. If the laser beam is circularly polarized, the angular momentum of the laser can drive the levitated particle to rotate^4–6^. By detecting the light passing through trapped particles, the motion of the trapped particle can be detected^7–9^. Therefore, an optical levitation, rotation and detection system can be realized by a single laser beam, which greatly simplifies the device system compared to other levitation methods, such as liquid^10^, air^11^, electromagnetic^12^, and electrostatic levitation^13^. Furthermore, the speed of the levitated particles can be driven to the GHz level by reducing the ambient air pressure^14–16^. Based on the optical levitation system^17^, high-precision measurements of force^18^, torque^14^, and acceleration^19^ have been realized. As the thermal motion of the trapped particles is cooled to the quantum ground state, the measurement accuracy is expected to further improve^20–22^.

High-speed rotating objects exhibit a stable spin axis in space, which can be used to measure angular motion. This phenomenon is known as the gyroscopic effect and has significant implications for engineering and navigation. Compared with vibration gyroscopes and optical gyroscopes, rotor gyroscopes have the longest history of development. However, their performance and accuracy have consistently remained at a superior level. The performance of a rotor gyroscope is known to increase with the speed of the rotating rotor; thus, there has been a persistent stimulus to realize a rotor gyroscope in high-speed rotating optical levitated particles. In the case of optically levitated rotating particles, gyroscopic stabilization has been observed and exploited in the MHz rotation of vaterite microspheres^23^ and the GHz rotation of silica nanodumbbells^5^. This property of rotating particles can be used to cool their rotations to sub-Kelvin temperatures^24,25^ and frequency-lock the rotation to an external drive, resulting in an ultrastable nanomechanical rotor^26^. On the other hand, because the trapping laser tends to align the major axis of the particle with the laser electric field, precession motion occurs in rotating nanoscale silica particles^24,27^. Precession can be used for torque sensing with a sensitivity capable of resolving single electron and nuclear spins^28^. Furthermore, novel strategies for precision tuning of the orientation of levitated microspheres have been proposed^29^, which are indispensable for the realization of micromotors and micro gyroscopes. Despite extensive research on the dynamics of optically levitated rotating rotors, theoretical and experimental studies on the measurement of angular motion in the presence of specific obstacles are lacking. First, the optical torque that aligns the particle optical axis with the laser electric field^27,28^ causes the rotor to process, which differs from traditional free mechanical rotor gyroscopes. In addition, measuring the spin axes of optically levitated particles is challenging due to their small volume.

Here, we successfully demonstrated the practical application of optical levitation technology in the measurement of external angular motion by overcoming these obstacles. In this paper, the gyroscopic dynamics of an optically levitated rotor are analyzed, with a focus on investigating the influence of the rotor’s optical axis and geometric long axis on the attitude angle. By considering the precession of a high-speed rotor, the gyroscope response is deduced when subjected to an external angular velocity. According to the theoretical model, an optical levitation system is built in a vacuum to realize ultrafast rotation of the trapped particle, and the gyroscopic effect is validated by detecting the spin axis according to the rotational signal. This represents the first reported implementation of an optically levitated gyroscope with a rotating microrotor.

## Results

### Working principle

The presented optically levitated gyroscope (OLG) is a type of rotor gyroscope that senses angular motion via the inertia of a high-speed rotating particle. Particle levitation, spin driving, and output detection are both realized by a single laser beam. In this section, a gyroscopic model is established to investigate the dynamic output of the OLG when the external angular motion is input. Then, a spin frequency-based detection method is presented to obtain the output signal.

**Fig. 1:**
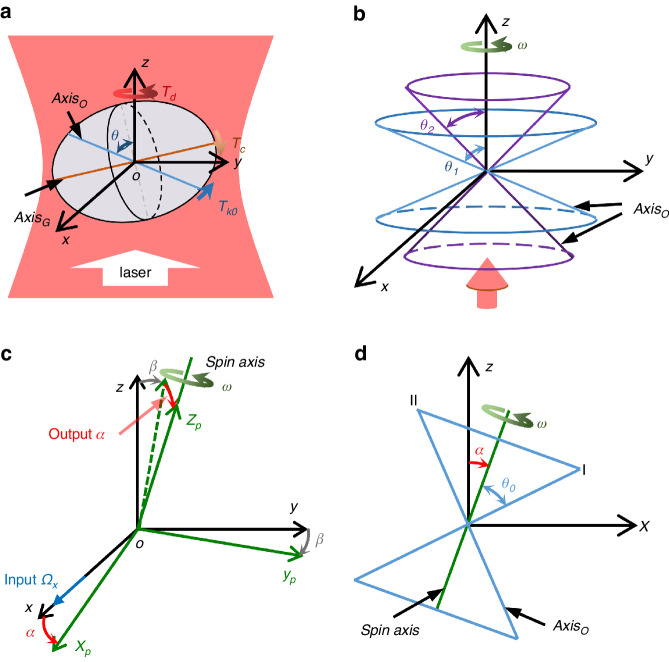
Dynamical model of the optically levitated rotor gyroscope. **a** An ellipsoidal birefringent particle with optical axis *Axis*_*O*_ and geometrical long axis *Axis*_*G*_. The centrifugal torque *T*_*c*_ comes from the rotation of the particle and is balanced with the restoring torque *T*_*k*0_. *T*_*d*_ is the driving torque. **b** Trajectory of the optical axis under rotation. Due to the rotation, the trajectory of the optical axis is cone-shaped. The color represents the different angles *θ*_*1*_ and *θ*_*2*_. **c** Coordinate definition of the gyroscope. *xyz* is the gyroscope case frame, and *x*_*p*_*y*_*p*_*z*_*p*_ is the rotor frame. *α* and *β* are the angular displacements of the rotor relative to the gyroscope case. *Ω*_*x*_ is the external angular velocity to be measured. **d** Side view of the spin axis with a deflection angle *α* and an angle *θ* = *θ*_*0*_ + *α* at position I and *θ* = *θ*_*0*_ − *α* at position II

As illustrated in Fig. 1a, an ellipsoidal birefringent particle is radiated by a circularly polarized laser propagating along the positive *z*-axis direction. The blue line, *Axis*_*O*_, in Fig. 1a, represents the optical axis of the birefringent particle, and the brown line, *Axis*_*G*_, represents the geometric long axis. Due to the nonideal spherical shape of particles, the optical axis and the geometric long axis of the particle typically do not align. The optical torque exerted by a circularly polarized laser can be decomposed into two components: a driving torque and a restoring torque^27–29^. The driving torque, *T*_*d*_, induces rotation of the particle, with the spin frequency being determined by the balance between the frictional torque from the surrounding air and the driving torque *T*_*d*_. On the other hand, the restoring torque, *T*_*k0*_, aligns the optical axis *Axis*_*O*_ of the ellipsoidal birefringent particle parallel to the transverse plane (the plane of the rotating electric field, *x-o-y* in Fig. 1a) of the laser. As a result, when the particle is not rotating, the angle *θ* between *Axis*_*O*_ and the laser propagation direction (*z*-axis) is approximately 90°, and the restoring torque *T*_*k0*_ is 0.

As the spin frequency of the particle increases gradually, due to geometric asymmetry, the centrifugal torque *T*_*c*_ (creating a heavier end to the rotation plane) also increases, making the optical axis deviate from the transverse plane^29^. When the spin frequency becomes stable, the centrifugal torque *T*_*c*_ and the restoring torque *T*_*k0*_ are balanced, and the angle *θ* is maintained at a specific position *θ*_*0*_ ≠ 90°. The restoring torque^27^ can be expressed as *T*_*k0*_∝sin(2*θ*)*E*^2^, where *E* represents the laser electric field intensity. When the particle oscillates slightly near the equilibrium position, the restoring torque can be approximated as linear *T*_*k*_ ≈ *k*Δθ, where *T*_*k*_ is the spring torque. This behavior can be analogized to a torsion spring with a stiffness coefficient of *k*, which limits the orientation of the optical axis to the vicinity of its initial position. The stiffness coefficient *k* is dependent on factors such as the particle shape, material, and laser power. Although the optical axis rotates with the particle rotation, the angle *θ* remains unchanged, as shown in Fig. 1b.

However, when an external angular velocity is applied, the dynamic response of the particle changes the angle *θ*. As shown in Fig. 1c, the coordinate frame *xyz* is fixed to the laser beam (gyroscope case), and the coordinate frame *x*_*p*_*y*_*p*_*z*_*p*_ is fixed to the particle (rotor). The particle spins around the o*z*_*p*_ axis, and the two coordinate frames coincide in the absence of inputted angular motion. When an external angular velocity *Ω*_*x*_ is applied to the laser beam in the *x* direction, the particle spin axis does not rotate simultaneously due to the isolation of the particle from the laser beam. When there is a relative angular displacement Δ*θ* = *β* between the laser beam (*z*) and the particle (*z*_*p*_), the particle is subjected to the abovementioned spring torque along the *x*-axis. In this case, the spinning particle would precess along the *y*_*p*_ axis, with a precession angle *α*. As a result of the deviation *α*, the spring torque along the *y*-axis makes the particle precess along the *x*-axis. This process reduces and eventually eliminates the angular displacement *β*. In the steady state, the particle precesses along the *x*-axis with *Ω*_*x*_, and the deflection angle *α* is read out to extract the external input angular velocity.

Assuming the spin frequency is *ω*, the moment of inertia is *I*, and the angular momentum of the particle is *H* = *Iω*. For an ideal circularly polarized Gaussian beam, the spring torque stiffness coefficients along the *x-* and *y*-axes are equivalent, given as *k*_*x*_ = *k*_*y*_ = *k*. Additionally, the impact of sphericity errors in particles on their rotational damping is considered negligible. Therefore, the damping coefficients can be approximated as equal, represented as *c*_*x*_ = *c*_*y*_ = *c*. The gyroscopic dynamic equation of the particle is established as^30–32^

1 $$\left\lbrace\begin{array}{lll} H {\dot\alpha} - c_{x} \dot\beta - k_{x} \beta = T_{th} \\H \dot\beta + c_{y} \dot\alpha + k_{y} \alpha = H \it\Omega_{x} + T_{th} \end{array}\right.$$

where *Hα’* and *Hβ’* are gyroscopic (precession) torques, *c*_*x*_*β’* and *c*_*y*_*α’* are damping torques, *k*_*x*_*β*, and *k*_*y*_*α* are spring torques, and *H*Ω_x_ is the equivalent gyroscopic torque resulting from the external input angular velocity. If the thermal fluctuation torque *T*_*th*_ is ignored, the solution to an input angular velocity *Ω*_*x*_ can be obtained (Supplementary Note 1). The rotation angle *β* is transient, while a steady deflection angle *α* = Ω_x_*H*/*k* will be generated in the orthogonal direction, and it is linear with the angular velocity of the input.

As shown in Fig. 1d, the input angular velocity can be detected by measuring the deflection angle *α* of the spin axis. The angle between the optical axis and the spin axis remains constant at *θ*_*0*_, while the angle *θ* between the optical axis and the laser propagation direction changes with *α*. Assuming that the initial position of the optical axis coincides with the *x*-axis (position I), the angle *θ* satisfies the following equation:

2 $$\cos \theta =\,\cos {\theta }_{0}\,\cos \alpha -\,\sin {\theta }_{0}\,\sin \alpha \,\cos (\omega t)$$

**Fig. 2:**
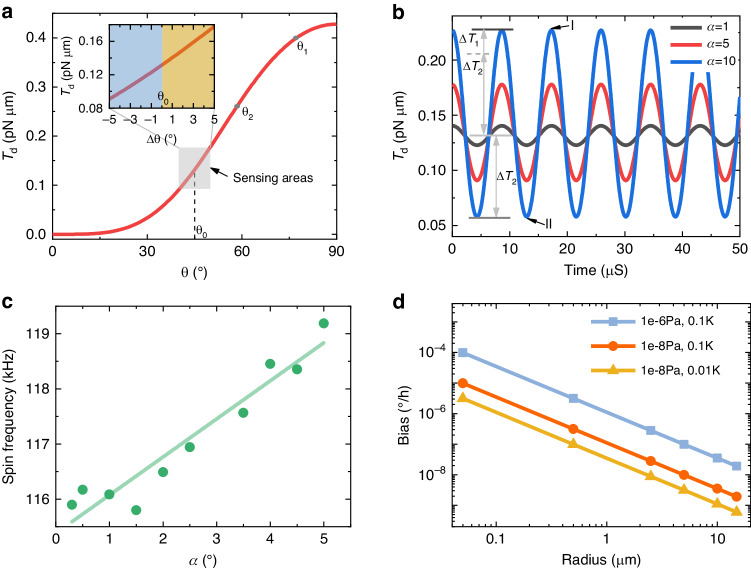
Dynamical response and performance of the optically levitated rotor gyroscope. **a** Driving torque *T*_*d*_ versus angle *θ*. The sensing areas indicate the gyroscope application analyzed above. The simulation parameters are presented in Supplementary Note 2. **b** Driving torque *T*_*d*_ versus time under different deflection angles *α*. *T*_*d*_ reaches maximum and minimum values at positions I and II, respectively. The average torque increases with *α*, and the fact that *ΔT*_*1*_ is larger than *ΔT*_*2*_ shows an increase in the average torque. The initial angle is *θ*_*0*_ = 45°, and the initial spin frequency is 116 kHz in the simulation. **c** Spin frequency versus deflection angle *α*. The nonlinear error comes from the numerical solution of differential Eq. (4). **d** Calculated bias instability of optically levitated gyroscopes with different parameters (see Supplementary Note 3)

When *θ* deviates, the polarization of the transmitted light changes. Since the driving torque *T*_*d*_ comes from the transfer of the spin angular moment of the laser, the changes in polarization result in a variation in *T*_*d*_. The driving torque is expressed as^29^

3 $${T}_{d}={c}_{1}\,\sin \left({c}_{2}\left(1-\frac{{n}_{e}}{\sqrt{{n}_{o}^{2}{\sin }^{2}\theta +{n}_{e}^{2}{\cos }^{2}\theta }}\right)\right)$$

where *c*_*1*_ and *c*_*2*_ are constant parameters related to the laser and particle parameters, respectively, and *n*_*e*_ and *n*_*o*_ are the refractive indices of the birefringent vaterite particle (see Supplementary Note 2 for details). As shown in Fig. 2a, when the angle is *θ* = 90°, the driving torque *T*_*d*_ reaches its maximum value. As the optical axis deviates from the transverse plane (decreasing from 90°), the driving torque *T*_*d*_ decreases. According to the relation between *T*_*d*_ and *θ*, the orientation *θ* of the optical axis can be obtained by monitoring the driving torque, while the driving torque can be measured by the spin rotational signal of the particles^29,33^.

Substituting Eq. (2) into Eq. (3), the driving torque *T*_*d*_ fluctuates, as shown in Fig. 2b, due to the changes in *θ*. The average driving torque increases with *α*, which results in a variation in the spin frequency. The rotational dynamics equation of spin is

4 $$\frac{d\omega }{dt}I={T}_{d}-c\omega$$

Substituting Eqs. (2) and (3) into Eq. (4), the spin frequency under different deflection angles *α* is shown in Fig. 2c. Since *α* is related to the input angular velocity *Ω*_*x*_, the spin frequency is hence regarded as the output of the gyroscope in the following experiment. It is worth noting that the changes in the driving torque are related to the initial angle *θ*_*0*_; hence, the sensitivity can be modulated by controlling the initial stable angle. The controlling methods include changing the spin frequency and regulating the particle manufacturing process^29,34,35^.

As shown in Fig. 2d, taking the thermal fluctuation torque into account in dynamic Eq. (1), the bias instability of this gyroscope can be estimated by the equivalent angular velocity *Ω*_*th*_=*T*_*th*_/*H* (Supplementary Note 3). The bias instability is *B* = *k*_*σ*_*Ω*_*th*_∝(*T*_*emp*_*P*_*0*_/*a*^3^)^1/2^, where *k*_*σ*_ is the bias instability conversion coefficient, *T*_*emp*_ is the torsional vibration equivalent temperature, *P*_*0*_ is the pressure, and *a* is the radius of the rotor. Therefore, the bias instability can be improved by decreasing the motion temperature^24^ and pressure or increasing the particle size, and an instability of 10^−9^°/*h* is possible for a 30 μm diameter particle with a torsional vibration equivalent temperature of 0.1 K under 10^−^^8^ Pa.

### Experimental verification

**Fig. 3:**
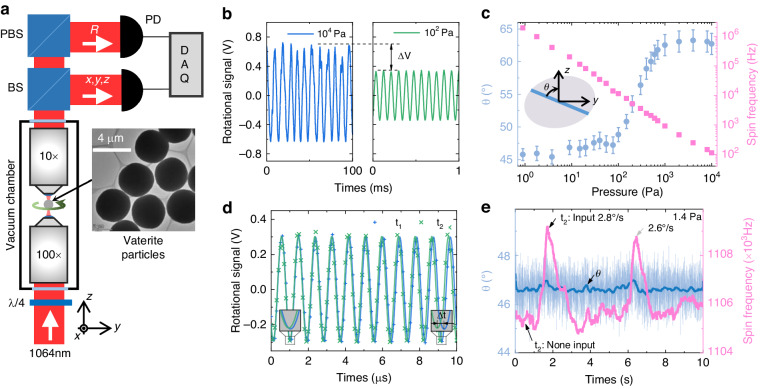
Experimental setup and the measurement results of alignment and gyroscopic effects. **a** Sketch of the experimental setup. The labels denote the quarter-wave plate (*λ*/4, used for obtaining the circularly polarized laser), microscope object (100× and 10×), beam splitter (BS), polarizing beam splitter (PBS), photodetector (PD), and data acquisition card (DAQ). The inset shows the vaterite particles used as a rotor. **b** Spin rotational signal of a trapped particle at different pressures, where the amplitude is related to *θ*. **c** *θ* and spin frequency versus pressure. *θ* decreases with decreasing pressure, which means that the optical axis rotates toward the propagation direction of the laser. **d** Spin rotational signal at different times *t*_1_ and *t*_2_, and the angular velocity is inputted at time *t*_2_. **e** Changes in *θ* and the spin frequency under the input angular velocity

An optical levitation system (Fig. 3a, see Methods section for details) is established to validate the proposed theoretical model. Vaterite particles with a mean diameter of 3.58 μm and a standard deviation of 0.19 μm are trapped in a vacuum and driven to spin at frequencies ranging from 100 to 1000 kHz. The spin and center of mass motion are measured by a photodetector and recorded by a data acquisition card. To introduce angular velocity and isolate the vibration from the ground, the setup is constructed on an anti-vibration air-floating platform.

As mentioned above, the initial position *θ*_*0*_ will change under the action of centrifugal torque during the process of spin rotation acceleration. Figure 3b shows two spin rotational signals of a particle at pressures of 10^4^ Pa and 10^2^ Pa. The spin frequency increases with decreasing pressure while the amplitude of the rotational signal decreases. According to the optical torque measurement method^29,33^, a larger amplitude represents a larger torque. Moreover, the angle *θ* is related to the torque, as shown in Fig. 1d; hence, a decrease in the amplitude indicates a corresponding change in *θ*. Since the spin frequency is also sensitive to pressure, the angle *θ* is measured by the amplitude when decreasing the pressure. Figure 3c displays the measured *θ* under different pressures, and the transform method from amplitude to *θ* is presented in Supplementary Note 2. With decreasing air pressure, the spin frequency increases gradually, resulting in an increase in the centrifugal torque, so the optical axis of the particles gradually moves away from the initial position. The significant decrease within 10^3^–10^2^ Pa is the result of a greater growth rate of the spin frequency, which is subtle for the logarithmic coordinates. When the pressure drops below 100 Pa, the polar angle tends to stabilize at approximately 46°, which means that the long axis is already close to the rotation plane.

When the pressure remains constant, the spin frequency is utilized to represent the deflection angle *α* induced by the input angular velocity. Figure 3d compares the spin rotational signal with (*t*_2_) and without (*t*_1_) the external input angular velocity. The amplitudes of the two signals are approximately equal due to the small deflection angle, while the periods exhibit inequality, indicating that the frequency is more sensitive to the external angular velocity. The time difference Δt confirms the changes in frequency. As shown in Fig. 3e, the frequency and amplitude of the spin rotational signal are obtained by fitting (see Methods), and the frequency changes by approximately 4 kHz when there is an external angular velocity of 2.8°/s. Since the deflection angle is approximately 0.5°, the variety of amplitudes is approximately 8 mV (smoothed line). However, the standard deviation of the amplitude is approximately 10 mV, which is beyond the variety.

### Gyroscope performance

**Fig. 4:**
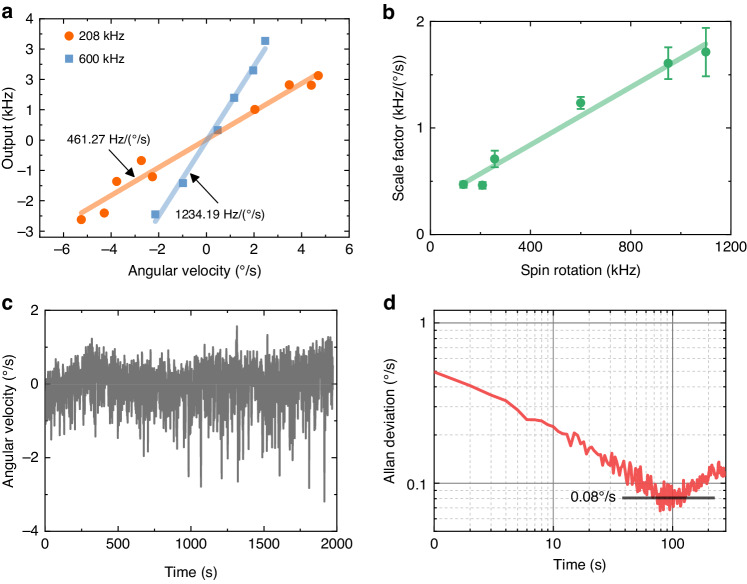
Gyroscopic performance of the optically levitated rotor gyroscope (OLG). **a** The output of the OLG under different external angular velocities. Extended data are presented in Supplementary Fig. S6. **b** Scale factor under different spin frequencies. The error bars are determined by fitting. **c** Measured angular velocity with no input, which shows the instability of the OLG. The spin frequency of the rotor is 470 kHz at 4 Pa, and the sampling rate is 1 Hz. **d** Allan deviation of the prototype, where the bias instability is 0.08°/s

The gyroscopic effect is evaluated under different stable spin frequencies (stable pressures). As shown in Fig. 4a, the change in the spin frequency roughly corresponds linearly to the input angular velocity. There are two main reasons for the inconsistency in the measurements. One is the transient response of *α* and *β* to the input angular velocity, and the other is the fluctuation in the spin frequency caused by changes in the experimental parameters. As shown in Fig. 4b, the response at different spin frequencies shows that the scale factor of the optically levitated gyroscope increases with increasing spin frequency, which is consistent with the theoretical results. The deflection angle of the gyroscope is *α* = Ω*H*/*k*. As the spin frequency of the particles increases, the angular momentum *H* increases, resulting in an increase in the sensitivity *H/k*. For example, the scale factor is 461.27 Hz/(^o^/s) when the spin frequency is 208 kHz, while the scale factor is 1234.19 Hz/(^o^/s) when the spin frequency increases to 600 kHz. The difference between the ratios of the scale factor (2.68) and spin frequency (2.88) is approximately 7%.

In addition, we also evaluated the bias instability of the optically levitated rotor gyroscope. The output signal without the external angular velocity for half an hour is presented in Fig. 4c. The output signal (i.e., the product of the spin frequency variation and scale factor) exhibits high-frequency fluctuations and long-period slow changes. The fluctuation around the mean value is mainly caused by the thermal motion of the particles, while the slow change is the result of the changes in pressure and laser power during the test. Furthermore, decreases in the output signal consisted of sharp drops, while increases were more moderate. For example, the output fluctuation reached −3°/s at 1800 s. This is because the thermal motion of the particles causes them to deviate from the laser focus, resulting in a reduction in the laser power. This reduction in laser power leads to a decrease in the spin frequency, ultimately affecting the stability of the test^36^. As shown in Fig. 4d, the Allan variance curve shows that the bias instability of the prototype is approximately 0.08°/s. By improving the above factors, such as decreasing the fluctuation in the laser power and cooling the particle, the stability of the optically levitated gyroscope can be improved^26,37^.

## Discussion

This paper represents the first operation and evaluation of a micro rotor gyroscope based on optically levitated particles. The working principle of this gyroscope is proposed theoretically according to the response of the spin axis to the input angular motion, and the gyroscopic effect is validated experimentally according to the measurement of the spin axis using the rotational signal. Furthermore, this paper describes the development of the dynamics and sensing applications of a levitated optomechanical system, and new development opportunities for traditional mechanical rotor gyroscopes are presented. At the same time, acceleration measurement techniques based on levitated particles are very mature^38,39^. By integrating these techniques with the angular velocity measurement scheme introduced in this study, a single levitated particle can complete the functions of both an accelerometer and a gyroscope to achieve inertial navigation. Compared to multiaxis combined inertial sensing technology, this scheme can greatly reduce the complexity of the system and is expected to become a new generation of high-precision measuring elements. Although the sensitivity and instability of the gyroscopes are not ideal at present, the performance of the gyroscopes can be greatly improved by laser cooling, customized levitated rotors, and enhanced optical trap stiffness^40–42^, thus providing new sensing technology for inertial navigation. These experiments are implemented in a free space optical system; however, the presented optically levitated gyroscope has the potential to be realized on a chip^43–45^, and hybrid methods can be used to calculate the optical force and torque in an integrated system^46,47^.

## Methods

### Experimental setup

**Fig. 5:**
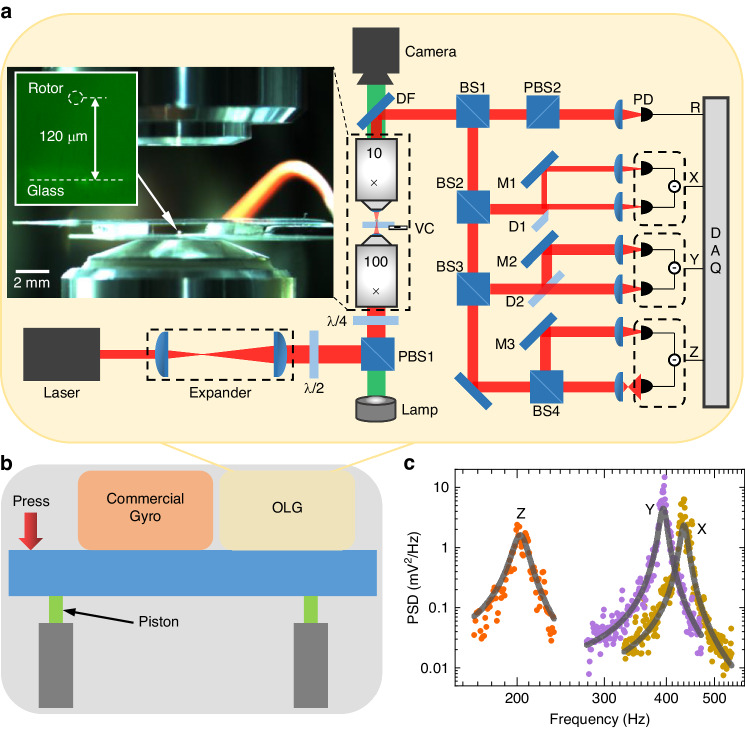
Experimental setup. **a** The labels denote the half-wave plate (*λ*/2), polarizing beam splitter (PBS), quarter wave plate (*λ*/4), dichroic filters (DF), beam splitter (BS), D-shaped mirror (D), mirror (M), photodetector (PD), and vacuum chamber (VC). The inset is a picture of the experimental setup. **b** Schematic diagram of the gyroscopic effect test. The optically levitated rotor gyroscope (OLG) and the commercial gyroscope are placed on the air-floating platform, and the angular motion of the air-floating platform is input by pressing the platform. **c** Power Spectrum Density (PSD) of the center of mass motion of a vaterite particle trapped by a circularly polarized laser at 40 Pa

As shown in Fig. 5a, b, the optically levitated rotor gyroscope and a commercial gyroscope (InvenSense ICM-20602) are both installed on the platform. The experimental optical path is presented in Fig. 5a, where the red and green lines denote the trapping and imaging light, respectively. A vertical optical levitation system is utilized in the experiment. The trapping laser (LASEVER, LSR1064NL, wavelength 1064 nm, power 300 mW) was focused by a high numerical aperture microscope (Nikon, E Plan ×100, NA = 1.25) in the vacuum chamber. The laser power used in the experiment was approximately 20 mW. Before the laser enters the vacuum chamber, the beam expander is used to expand the beam diameter to approximately 1.5 times that of the pupil behind the objective lens, thereby maximizing the focusing ability of the objective lens and enhancing the trap stiffness. The laser power is then adjusted by the combination of a 1/2 wave plate and a polarizing beam splitter (PBS1), with PBS1 also adjusting the laser polarization. The extinction ratio of linearly polarized light from the PBS, TP:TS, exceeded 3000:1. This configuration, combined with the subsequent 1/4 wave plate, ensures a high degree of circular polarization of the laser entering the vacuum chamber.

The laser is focused by a high numerical aperture objective in the vacuum chamber, and particles are trapped near the focus. The vibration and rotation of the trapped particles will modulate the laser passing through the particles. The transmitted light is collected by a low-NA objective lens and reflected by the dichroic mirror to the detection part. In this case, the transmitted light is divided into four beams to detect the particle rotation signal (R) and three-dimensional vibration signal (*xyz*). The polar angle of the particle’s optical axis is determined by the amplitude or frequency of the rotational signal. The rotational signal (polarization modulated) of particles is distinguished after passing through PBS2 so that the signal of rotation motion can be read out by detecting its power changes with a photodetector. The vibration signals of the *xyz* axes are obtained via differential methods. The movements in the *x* and *y* directions are distinguished by D-shaped mirrors in different directions, and the movement in the *z* direction changes the focus position of the beam by the refraction of particles, resulting in a change in the light intensity on the probe. As shown in Fig. 5c, the resonant frequencies of the trapped particle are approximately *f*_*x*_ = 430 Hz, *f*_*y*_ = 390 Hz, and *f*_*z*_ = 200 Hz.

### Sample preparation

The particles utilized in the experiment consist of a birefringent material known as vaterite, which is synthesized via a solution-based method. These particles have a mean diameter of 3.58 μm and a standard deviation of 0.19 μm, and their shape and elemental mapping are clearly delineated in Fig. S5. In the experiment, the particles are placed in a small chamber (designed without sealing to ensure adequate ventilation and reduce the impact of airflow disturbance on the particles), and the small chamber is installed on the piezoelectric plate. Through the vibration of the piezoelectric plate, the particles are thrown near the laser focus to achieve trapping.

### Gyroscopic effect experiments

The comparative results of the optically levitated gyroscope and the commercial gyro are shown in Fig. S6. The output of the OLG is the change in the spin frequency of the levitated rotor. The spin frequency is obtained by fitting the rotational signal, as shown in Fig. S7a, and it is fitted for every 1 ms. Since the frequency resolution of the Fourier transform is 1/*T*, where *T* is the sampling time, the frequency space is 1 kHz for 1 ms. Nevertheless, the frequency resolution of the fitting is more precise. As shown in Fig. S7b, we use the signal generator to test the resolution of the fitting method. The initial output frequency is 100 kHz, and then the frequency is changed step by step (10 mHz). It is clear that the frequency resolution of the fitting method is less than 10 mHz.

### Supplementary information

Theoretical model and bias instability analysis of the proposed optically levitated gyroscope. Optical axis measurement method for birefringent particles. Information regarding the gyroscopic effect experiments and particles that were used in this work.

### Supplementary information

supplements
