# Supplementary material for: Optically levitated micro gyroscopes with an MHz rotational vaterite rotor
Source: Microsyst Nanoeng. 2024 Jun 18;10:78. doi: 10.1038/s41378-024-00726-0 (PMC11183073; doi:10.1038/s41378-024-00726-0)
Supplement: Supplementary file 1 — supplements [file 41378_2024_726_MOESM1_ESM.docx]

**Supplement: Optically levitated micro gyroscopes with a MHz rotational vaterite rotor**

Kai Zeng^1^, Xiangming Xu^2^, Yulie Wu^1^*, Xuezhong Wu^1^, Dingbang Xiao^1^*

*^1^ College of Intelligence Science and Technology, National University of Defense Technology, Changsha 410073, China.*

*^2^ College of Information and Communication, National University of Defense Technology, Wuhan 430000, China.*

* Corresponding author. Email: ylwu@nudt.edu.cn, dingbangxiao@nudt.edu.cn

1. Supplementary Note 1: Theoretical model

Fig. S1a shows the definition of the coordinate system of optically levitated particles, the coordinate origin *o* is the center of particles, and the *xyz* axes are the radial direction of particles. In the following, *I_j_* (*j*=*x*, *y*, *z*) represents the moment of inertia, *k_j_* represents the optical torsion spring stiffness ^1-3^, *c_j_* represents the particle rotational damping coefficient, and Ω_j_ represents the input angular velocity, *α* and *β* represents the rotational angle of particles along the *y* and *x* axes, respectively. The positive rotation direction of *α* is consistent with the *y*-axis, but *β* is opposite to the *x*-axis. As shown in Fig. S1b, the direction of phase plane *α-o'-β* can be consistent with the direction of *xy* with the above definition of α and β. It is assumed that the circularly polarized laser propagates along the positive direction of the *z* axis, and the particle rotates around the *z* axis under the driving torque with angular frequency *ω* , the angular moment of particles is *H*=*I_z_ω.*

- - - 1. **Definition of Coordinate system.** (**a**) Sketch map of rotational angle *α* and *β*, external angular velocity *Ω_x_* and *Ω_y_*, angular moment *H*. (**b**) Phase plane representation of particle rotation. The blue part is used for vision.

The theoretical model is presented as shown in Fig. S2. Since this paper mainly discusses the rotational motion of the particle spin axis, only the torque acts on the particle is analyzed. In the direction of *x*-axis, the particle is subjected to the thermal noise torque *T_th_*, and the torque corresponding to the angular acceleration *-I_x_Ω'x*, *I_x_β''*, gyroscopic torque *-HΩ_y_*, *-Hα'*, damping torque *c_x_β'*, restoring torque *k_x_β*.The torque in the *y*-axis is similar to that, so the dynamic equation is established ^4-6^

- - - 1. **Sketch map of the gyroscope working principles.** (**a**) Theoretical model for dynamic analysis. When there is only an angular velocity *Ω_x_* in *x* axis, the particle would follow the rotation in the direction of input angular velocity as (**c**), while the orthogonal direction would produce a deviation *α* as (**b**). *TP* represents the transverse plane of laser.

 (S1)

For an ideal circularly polarized Gaussian beam, the stiffness coefficients of particles in different directions are equal *k_x_*=*k_y_*=*k*. The influence of sphericity error of particles on their rotational damping is also slight, so the damping coefficient can also be approximately equal to *c_x_*=*c_y_*=*c*. Assuming that the input angular velocity is a constant value, the torque corresponding to the angular acceleration *-I_x_Ω'_x_* can be ignored. The torque *I_x_β''* corresponding to the nutation of the particle spin axis can also be ignored because its frequency is twice the rotation and its amplitude is small. It is further simplified as that there is angular velocity *Ω_x_* input in the *x*-axis direction only, and the thermal noise torque is ignored. The simplified motion equation of the optically levitated rotor is

 (S2)

The equation is solved as

$\left\{\begin{matrix} \alpha=\frac{H}{k}\Omega_{x}-\frac{H}{k}\Omega_{x}\exp\left(\frac{-ck}{H^{2}+c^{2}}t\right)\cos\left(\frac{kH}{H^{2}+c^{2}}t\right) \\ \beta=\frac{H}{k}\Omega_{x}\exp\left(\frac{-ck}{H^{2}+c^{2}}t\right)\sin\left(\frac{kH}{H^{2}+c^{2}}t\right) \end{matrix}\right.$ (S3)

It can be seen that when there is an external angular velocity in the *x*-axis, the spin axis of particles will respond around the *x*-axis and *y*-axis simultaneously. But in the same direction, the angle *β* (rotation about the *x*-axis) is an oscillating rotation (libration) with attenuation. Although there is also an oscillation term about *α* along the *y*-axis, it will eventually produce a stable deflection angle *Ω_x_H/k*. In other words, when the angular velocity *+Ω_x_* is input along the positive direction of *x* axis (Fig. S2c), the particle would rotate in the positive direction of *y* axis (Fig. S2b).

The specific change rule is shown in Fig. S3a, and the angular velocity of 2 ^o^/s is input along the positive direction of the *x*-axis at zero time. It can be found that the particle rotate along the *x* and *y* axes simultaneously, but the rotation along the *x* axis gradually tends to 0, and the rotation along the *y* axis gradually stabilizes at a fixed angle. The motion track of the spin axis endpoint is shown in Fig. S3b, it can be seen that the spin axis finally stabilized in a spiral motion.The spiral motion of the spin axis is mainly caused by the restoring torque and damping torque. As shown in formula (S3), the angular frequency of spiral rotation is *kH/(H^2^+c^2^)*, and the attenuation constant of rotation amplitude is *ck/(H^2^+c^2^)*.

- - - 1. **Simulation results of the rotational angle.** (**a**) Rotation response to the input angular velocity *Ω_x_*. (**b**) Motion track of particle spin axis endpoint. The spin rotation frequency is 1MHz, and the rotor is a 4μm diameter vaterite spherical particle.

From the above analysis, it can be found that after inputting angular velocity in a certain direction, a stable angle will be generated in its orthogonal direction. Due to the existence of the angle, the rotor is subjected to a restoring torque, which makes the rotor generate the precession following the input angular velocity, thus reaching a stable state. In this state, by detecting the deflection angle *α=Ω_x_H/k* in the orthogonal direction, the input angular velocity *Ω_x_* can be obtained, thus realizing the measurement of external rotation by optically levitated particles.

1. Supplementary Note 2: Optical axis measurement

The measurement of the deflection angle is realized by detecting the rotational signal under different driving torque as shown in Fig. 1d. The driving torque *T_d_* is ^3^

$T_{d}=\frac{PB_{0}}{\upsilon}\hat{z}\sin^{2}\left(\frac{2\pi an_{o}}{\lambda}\left(1-\frac{n_{e}}{\sqrt{n_{0}^{2}\sin^{2}\theta+n_{e}^{2}\cos^{2}\theta}}\right)\right)$ (S4)

where *P* is the laser power, *B_0_* is the overlap between the trapping beam and the particle, *a* is the particle radius, υ and λ are frequency and wavelength of the laser separately, *ẑ* is the unit vector in *z* direction, *n_e_* and *n_o_* are the refractive indices for laser polarized along the extraordinary and ordinary axes. The simulation results in Fig. 1d in the main text used *P*=20mW, *B_0_*=0.05, *λ*=1064nm, *n_e_*=1.65 and *n_o_*=1.55.

The rotational signal is^3^

$V=\frac{T_{d}\upsilon G}{4}\cos\left(2\omega t\right)$ (S5)

where G=3kV/W is the conversion gain of the photo detector in our experiment. According to the measured amplitude of the rotational signal and the experimental parameters mentioned above, the angle *θ* can be obtained as shown in Fig. 3.

1. Supplementary Note 3: Bias instability analysis

When there is no angular velocity, but only the thermal noise torque is considered, the dynamic equation of the rotating particle can be expressed as

 (S6)

It can also be solved that the steady-state solution is

$\left\{\begin{matrix} \alpha_{th}=\frac{T_{th}}{k} \\ \beta_{th}=\frac{T_{th}}{k} \end{matrix}\right.$ (S7)

The thermal fluctuation torque acts on the particle in unit time can be expressed as ^7,8^

$T_{th}=\sqrt{4k_{B}T_{emp}I\gamma}$ (S8)

where *k_B_* is the Boltzmann constant, *T_emp_* is the ambient temperature, and *γ*=*c*/*I*=1/*τ* is the rotational friction coefficient of particles, which can be obtained by measuring the decay time *τ*. The thermal fluctuation torque is equivalent to the input angular velocity *Ω_th_*,

$\Omega_{th}=\frac{T_{th}}{H}$ (S9)

Since

$H=I\omega$ (S10)

and the max rotational frequency *ω_max_* is limited by the ultimate tensile strength *σ=7GPa* of the material ^9,10^, the max rotational frequency is ^11^

$\omega_{\max}=\sqrt{\frac{\sigma}{K\rho}}\frac{1}{a}$ (S11)

where *K=0.398* is a shape- and material-dependent constant, *ρ=2930kg/m^3^* is the density of vaterite, *a* is the radius of the spherical rotor.

The bias instability *B* can be estimated by the equivalent angular velocity

$B=k_{\sigma}\Omega_{th}=k_{\sigma}\sqrt{\frac{15k_{B}K}{8\pi\sigma}}\sqrt{\frac{T_{emp}\gamma}{a^{3}}}\propto\sqrt{\frac{T_{emp}P_{0}}{a^{3}}}$ (S12)

where *k_σ_*=0.304 is the bias instability conversion coefficient of the Standard Deviation Method ^12^. For the spherical vaterite particles with a diameter of 3.58 μm used in the experiment, the thermal noise equivalent bias instability is 0.005^o^/s when the rotating speed reaches 470kHz at 4Pa without cooling. The measurement results 0.08^o^/s is one order of magnitude larger than the calculated value for the disadvantageous reasons, such as center of mass motion that can be decrease by laser cooling, laser power and environment temperature stability that can be suppressed by improving experimental equipment. As shown in Fig. S4, the instability of the optically levitated rotor gyroscope can be further improved by decreasing the motion temperature and pressure or increasing the particle size.

- - - 1. **Thermal noise stability of optically levitated gyroscope** under (**a**) different pressure and (**b**) particle size in 10^-8^ Pa.

1. Supplementary Figures

- - - 1. **Shape and element of the vaterite particles.** (**a**) Transmission Electron Micro-graph of vaterite particles. (**b**) Elemental mapping of one particle.

- - - 1. Gyroscopic effect of optically levitated particles with different rotational frequency (OLG: Optically Levitated Gyroscope). Since the stability of the spin axis is related to the spin frequency, the results consistency between OLG and commercial gyro is better for the higher frequency.

- - - 1. **Measurement method of the instantaneous rotational frequency.** (**a**) Raw and fitted data of the rotational signal of a levitated rotor with the rotational frequency 46.7kHz. (**b**) The changes of the fitted frequency, and the initial frequency is 100kHz.

**References:**

^1^ Li, M. *et al.*, Optical trapping force and torque on spheroidal Rayleigh particles with arbitrary spatial orientations. *Journal of the Optical Society of America A* **33** 1341-1347 (2016).

^2^ Rashid, M., Toros, M., Setter, A. & Ulbricht, H., Precession Motion in Levitated Optomechanics. *Phys Rev Lett* **121** 253601.1-253601.6 (2018).

^3^ Xie, S., Sharma, A., Romodina, M., Joly, N. Y. & Russell, P. S. J., Tumbling and anomalous alignment of optically levitated anisotropic microparticles in chiral hollow-core photonic crystal fiber. *Sci. Adv.* **7** (2021).

^4^ Hai, L., Liu, X., Chen, W. & Zhang, H., A miniaturized two-DOF rotational gyro with a ball-joint supported permanent magnet rotor. *Appl Phys Lett* **109** 07E525-514 (2016).

^5^ K., P. & U., W., Static Behavior of Closed-Loop Micromachined Levitated Two-Axis Rate Gyroscope. *Ieee Sens J.* **15** 7001-7008 (2015).

^6^ K., V. P., J., G. K. & V., B., Mechanical Thermal Noise in Micro-Machined Levitated Two-Axis Rate Gyroscopes. *Ieee Sens J.* **18** 1390-1402 (2018).

^7^ Haiberger, L., Weingran, M. & Schiller, S., Highly sensitive silicon crystal torque sensor operating at the thermal noise limit. *Rev Sci Instrum* **78** 025101 (2007).

^8^ Ahn, J., Xu, Z., Bang, J., Ju, P. & Li, T., Ultrasensitive torque detection with an optically levitated nanorotor. *Nat. Nanotechnol.* **15** (2020).

^9^ Ahn, J. *et al.*, Optically Levitated Nanodumbbell Torsion Balance and GHz Nanomechanical Rotor. *Phys Rev Lett* **121** 033603.1-033603.5 (2018).

^10^ Crist, B., The Ultimate Strength and Stiffness of Polymers. *Annual Review of Materials Science* **25** 295-323 (1995).

^11^ Schuck, M., Steinert, D., Nussbaumer, T. & Kolar, J. W., Ultrafast rotation of magnetically levitated macroscopic steel spheres. *Sci. Adv.* **4** e1701519 (2018).

^12^ IEEE Standard Specification Format Guide and Test Procedure for Coriolis Vibratory Gyros. *IEEE Std 1431-2004* 1-78 (2004).
